# A cross-sectional, multicenter survey of the prevalence and risk factors for Long COVID

**DOI:** 10.1038/s41598-022-25398-6

**Published:** 2022-12-27

**Authors:** Waki Imoto, Koichi Yamada, Ryota Kawai, Takumi Imai, Kengo Kawamoto, Masato Uji, Hidenori Kanda, Minoru Takada, Yoshiteru Ohno, Hiroshi Ohtani, Manami Kono, Atsuhito Hikiishi, Yosuke Eguchi, Hiroki Namikawa, Tomoya Kawaguchi, Hiroshi Kakeya

**Affiliations:** 1 Department of Infection Control Science, Osaka Metropolitan University Graduate School of Medicine, 1-4-3, Asahi-Machi, Abeno-Ku, Osaka, 545-8585 Japan; 2 Department of Infectious Disease Medicine, Osaka Metropolitan University Hospital, 1-5-7 Asahi-Machi, Abeno-Ku, Osaka, 545-8586 Japan; 3 Department of Infection Control and Prevention, Osaka Metropolitan University Hospital, 1-5-7 Asahi-Machi, Abeno-Ku, Osaka, 545-8586 Japan; 4 Department of Medical Statistics, Osaka Metropolitan University Graduate School of Medicine, 1-4-3, Asahi-Machi, Abeno-Ku, Osaka, 545-8585 Japan; 5 Department of Respiratory Medicine, Osaka City Juso Hospital, 2-12-27, Nonakakita, Yodogawa-Ku, Osaka, 532-0034 Japan; 6 Department of Internal Medicine, Kinshukai Hanwa The Second Hospital, 2-4-5, Abiko Nishi, Sumiyoshi-Ku, Osaka, 558-0015 Japan; 7 Department of Internal Medicine, Kinshukai Hanwa The Second Senboku Hospital, 3176, Fukai Kitamachi, Naka-Ku, Sakai-City, Osaka, 559-8271 Japan; 8 Department of Internal Medicine, Ohno Memorial Hospital, 1-26-10, Minamihorie, Nishi-Ku, Osaka, 550-0015 Japan; 9 Department of Surgery, Ohno Memorial Hospital, 1-26-10, Minamihorie, Nishi-Ku, Osaka, 550-0015 Japan; 10 Department of Respiratory Medicine, BellLand General Hospital, 500-3, Higashiyama, Naka-Ku, Sakai-Shi, Osaka, 599-8247 Japan; 11 Department of Medical Education and General Practice, Osaka Metropolitan University Graduate School of Medicine, 1-4-3, Asahi-Machi, Abeno-Ku, Osaka, 545-8585 Japan; 12 Department Respiratory Medicine, Osaka Metropolitan University Graduate School of Medicine, 1-4-3, Asahi-Machi, Abeno-Ku, Osaka, 545-8585 Japan; 13 Research Center for Infectious Disease Sciences, Osaka Metropolitan University Graduate School of Medicine, 1-4-3, Asahi-Machi, Abeno-Ku, Osaka, 545-8585 Japan

**Keywords:** Diseases, Risk factors, Signs and symptoms

## Abstract

Long-term sequelae of the coronavirus disease (COVID-19) constitute Long COVID. Although Long COVID has been reported globally, its risk factors and effects on quality of life (QOL) remain unclear. We conducted a cross-sectional study using questionnaires and electronic medical records of COVID-19 patients who were diagnosed or hospitalized at five facilities in Japan. Responses were obtained from 285 out of 1,150 patients. More than half of the participants reported Long COVID symptoms of varying severity 1 year after COVID-19. Common sequelae included fatigue, dyspnea, alopecia, concentration problems, memory problems, sleeplessness, and joint pain, which often significantly reduced their QOL. COVID-19 severity was strongly associated with sputum production, chest pain, dyspnea, sore throat, and diarrhea, but not with fatigue, dysgeusia, anosmia, alopecia, and sleeplessness. Fatigue, dysgeusia, anosmia, alopecia, and sleeplessness affected the QOL among participants with asymptomatic or mild COVID-19 during the acute phase. Moreover, these sequelae persisted for prolonged periods.

## Introduction

Coronavirus disease (COVID-19) presents with various symptoms, ranging from no symptoms in asymptomatic cases to severe pneumonia in acute cases. COVID-19 is associated with numerous complications including acute respiratory distress syndrome, thrombosis, heart disease, and other infectious diseases^1–9^. It also presents with long-term residual symptoms, known as post-acute COVID-19, post-COVID-19, or Long COVID^10–13^. Long COVID symptoms, including fatigue, dyspnea, joint pain, cough, dysgeusia, anosmia, headache, sputum production, and diarrhea^14–19^, are highly variable and differ according to their occurrence and the time elapsed from their onset^14–19^. In some studies, Long COVID sequelae were observed in 35–87% of participants up to 6 months post-onset^14–17^. However, there is insufficient research on the risk factors for each symptom and the effects of long-term residual symptoms of COVID-19 on quality of life (QOL). A large-scale investigation of Long COVID has not been conducted in Japan. Therefore, we conducted a multicenter, cross-sectional study on Long COVID and its risk factors among Japanese individuals. We have also discussed the real-world effects of long-term COVID-19 sequelae to help communities take the appropriate protective measures.

## Methods

### Study design

This was a cross-sectional questionnaire-based study.

### Eligibility criteria and study setting

**Figure 1:**
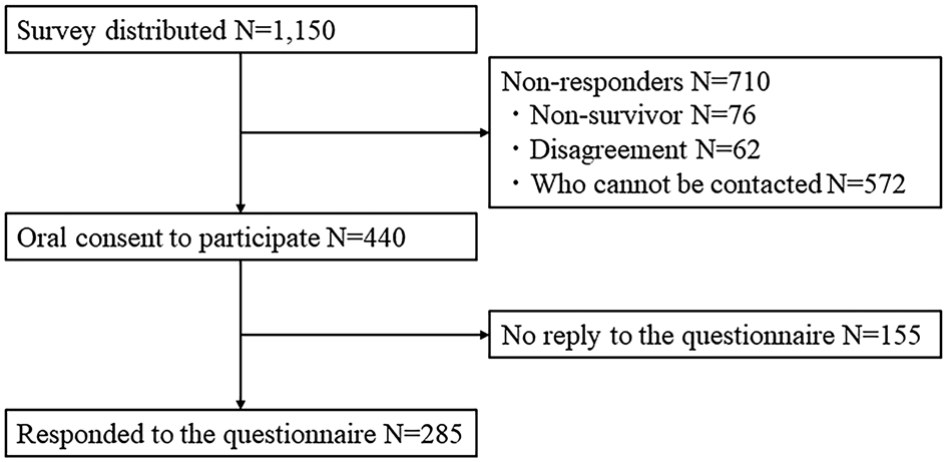
Patient selection for cross-sectional study.

This study was conducted at Osaka Metropolitan University Hospital, Osaka City Juso Hospital, Hanwa, The Second Hospital, Ohno Memorial Hospital, and Bell and General Hospital. All hospitals are medical institutions in Osaka, Japan. All patients diagnosed with SARS-CoV-2 infection (using polymerase chain reaction [PCR], a rapid antigen test, or loop-mediated isothermal amplification [LAMP]) or hospitalized with COVID-19 at each hospital between January 1, 2020 and December 31, 2020, were invited to participate (N = 1150). Their participation was confirmed over the telephone. Patients who did not or could not agree to participate, those who could not be contacted, and non-survivors were excluded. The questionnaire was sent to individuals who provided verbal consent regarding participation in September 2021, and those who returned the questionnaire within 1 month were included in the analysis (Fig. 1).

### Data collection methods

The following clinical data of participants who completed the questionnaire were collected from the electronic medical records of each hospital: age, sex, underlying disease, body height and weight, test used for microbiological confirmation of COVID-19, dates of admission and discharge, interval from COVID-19 diagnosis to completing questionnaire, severity of COVID-19 (asymptomatic, mild, moderate I, moderate II, and severe), outcome, and blood test results (white blood cell, lymphocyte, and platelet counts; hemoglobin, albumin, aspartate aminotransferase, alanine aminotransferase, creatinine, sodium, potassium, lactate dehydrogenase, creatine kinase [CK], creatine kinase myocardial band [CK-MB], C-reactive protein, D-dimer, fibrin/fibrinogen degradation products, ferritin, troponin-T, and procalcitonin levels; and prothrombin time). The severity of COVID-19 was defined according to the Ministry of Health, Labour and Welfare criteria, as follows^20^: mild: symptomatic but no pneumonia or dyspnea, no need for oxygen (corresponding to National Institutes of Health [NIH] category^21^ of “mild illness”); moderate I: with dyspnea or pneumonia but without the need for oxygen (corresponding to the NIH category of “moderate illness”); moderate II: requiring oxygen administration but no intensive care (corresponding to the NIH category of “severe illness”); and severe: intensive care unit treatment or mechanical ventilation required (corresponding to the NIH category of “critical illness”). In the analysis, participants were assigned to two groups based on the severity of their acute illness. Those with asymptomatic or mild disease were assigned to the Mild group, and those with moderate I, moderate II, or severe disease were assigned to the Severe group.

### Questionnaire

Participants were asked the following information: underlying disease (in those with missing information in electronic medical records), pregnancy at the time of infection (yes or no), smoking history (number of cigarettes and years), alcohol consumption (non-drinker, occasional drinker, drinking several times a week, drinking daily), activities of daily living (independent [self-reliant], some assistance required, completely dependent), symptoms present before infection, symptoms in the acute phase of COVID-19, and persisting symptoms at the time of answering the questionnaire. The symptoms at the time of answering the questionnaire, excluding symptoms that were present before COVID-19, were considered sequelae. The following symptoms were investigated: cough, sputum production, chest pain, fatigue, dyspnea, dysgeusia, anosmia, lack of appetite, sore throat, alopecia, concentration problems, memory problems, sleeplessness, dizziness, joint pain, red eyes, headache, and diarrhea. The severity of symptoms was graded as follows: **0**, no symptoms; **1**, occasional or intermittent symptoms but no treatment required; **2**, symptoms sometimes occur and interfere with activities of daily living; **3**, symptoms occur frequently and interfere with activities of daily living and concentration; and **4**, symptoms are always present. Participants also reported the time taken for symptom resolution.

### Statistical methods

Data from all participants were analyzed. The demographics and clinical characteristics were expressed as counts (percentage [%]) for categorical variables, and as medians (interquartile range [IQR]) for continuous variables. The percentage of participants with one or more symptoms during the acute phase of COVID-19, or at the time of answering questionnaire, was estimated with 95% confidence intervals using the Clopper–Pearson interval. The percentage of participants with increasing severity (0–4) of their respective symptoms was also summarized. An exploratory analysis was performed using univariable ordinal logistic regression models to assess the association between the symptom severity and variables measured in the acute phase of COVID-19. The odds ratio (OR) for high severity was used to express the relationship strength. The interval from COVID-19 onset to symptom disappearance was estimated for each symptom using the Kaplan–Meier method. The percentage of participants with each symptom at > 3 months, 6 months, and 1 year after COVID-19 onset was calculated. All analyses were conducted using R, Version 4.1.0 (R Foundation for Statistical Computing, Vienna, Austria).

### Ethics declarations

This study was approved by the Ethics Committee of the Osaka Metropolitan University Graduate School of Medicine (No. 2020-270) and all the ethics committees of the participating hospitals. All methods were performed in accordance with the relevant guidelines and regulations.

### Consent to participate

Verbal consent was obtained via telephone from the participants to confirm their willingness to participate after a thorough explanation of the purpose, risks, and benefits of the study. Additionally, the consent form, enclosed with the questionnaire and explanatory document about research purpose, risks, and benefits, was returned to us with the completed questionnaire, and informed consent was obtained from the participants.

## Results

Across the five participating hospitals, 1,150 patients tested positive for severe acute respiratory syndrome coronavirus 2 (SARS-CoV-2) by PCR, LAMP, or a rapid antigen test during the study period. Among them, 76 patients died, and 572 could not be contacted. Sixty-two patients did not agree to participate in the study. The remaining 440 patients provided verbal consent over the telephone to participate in the study. Questionnaires were sent to these 440 patients, of whom 155 did not reply, leaving 285 participants, a response rate of 24.8% (285/1,150) (Fig. 1).

**Figure 2:**
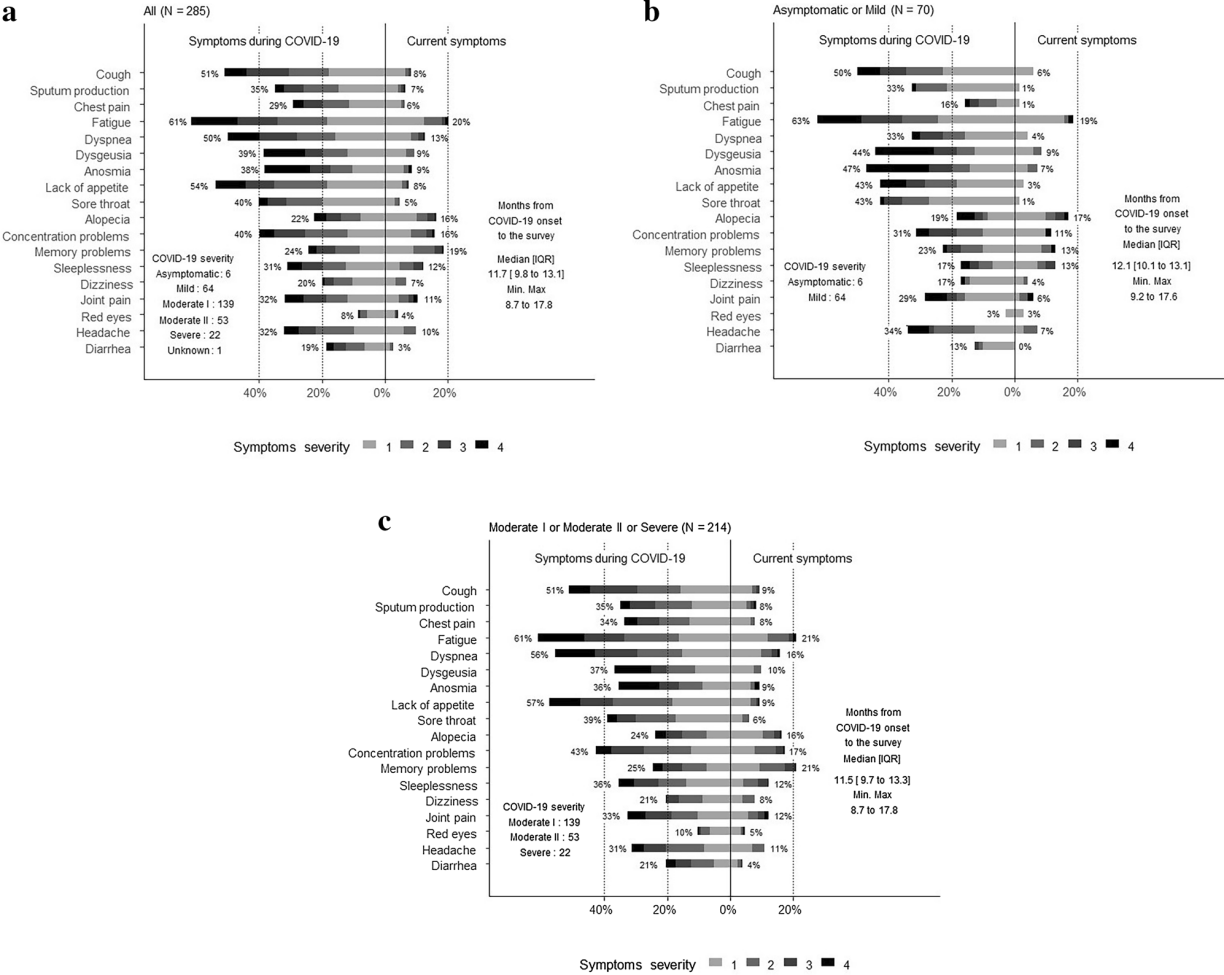
Distribution of each symptom in the acute phase and at the time of completing the questionnaire (**a**) All participants; (**b**) Mild group (asymptomatic and mild COVID-19 severity); and (**c**) Severe group (moderate I/II severity and severe COVID-19).

Participant demographics, medical history, and laboratory test results are shown in Table 1. The median age of participants was 60 years, and 57.2% were male. According to the Ministry of Health, Labour and Welfare criteria^20^, 2.1% of participants had asymptomatic disease, 22.5% had mild disease, 48.8% had moderate I disease, 18.6% had moderate II disease, and 7.7% had severe disease. According to COVID-19 severity, 70, 214, and 1 patients were assigned to the Mild, Severe, and Unknown groups, respectively. Hypertension was the most common underlying disease and was present among 29.5% of participants. Further, 17.9% of participants had diabetes, and 15.1% had dyslipidemia. The median time from COVID-19 onset to questionnaire completion was 357 days. At the time of the survey, 56.1% of participants had one or more COVID-19 sequelae. Of patients in the Mild and Severe groups, 52.9% and 57.5% had one or more sequelae, respectively (Table 2). Symptoms during the acute phase and at the time of answering the questionnaire are summarized in Fig. 2, which includes the severity of all sequelae. 20% of participants reported persistent fatigue, and ≥ 10% of participants reported persistent dyspnea, alopecia, concentration problems, memory problems, sleeplessness, joint pain, and headache at the time of answering the questionnaire. The symptoms experienced by participants in the acute phase included fatigue in ≥ 60%; cough, dyspnea, and lack of appetite in > 50%; and sore throat and concentration problems in 40% (Fig. 2a). Similarly, the distribution of symptoms in the acute phase and at the time of answering the questionnaire is shown for the Mild (Fig. 2b) and Severe groups (Fig. 2c). Among participants in the Mild group, the most common symptoms reported in the acute phase were cough, fatigue, dysgeusia, anosmia, lack of appetite, and sore throat; and the most common symptoms reported at the time of answering the questionnaire were fatigue and alopecia, followed by memory problems, sleeplessness, and concentration problems (Fig. 2b). Among participants in the Severe group, the most common symptoms reported in the acute phase were cough, fatigue, dyspnea, lack of appetite, and concentration problems, were (> 40% of participants); and the most common symptoms reported at the time of answering the questionnaire were fatigue, dyspnea, dysgeusia, alopecia, concentration problems, memory problems, sleeplessness, joint pain, and headache (≥ 10% of participants).

**Table 1:** Characteristics of study participants (N = 285).

	Median (IQR) or count (%)	n missing
Days from COVID-19 onset to the survey	357 (299–400)	0
Age (years)	60 (46–76)	0
Male	163 (57.2%)	0
Female	122 (42.8%)	0
Body mass index (kg/m^2^)	23.4 (21.5–26.4)	3
Smoker	134 (48.2%)	7
Alcohol drinker	210 (74.7%)	4
Drinking daily	79 (27.7%)	
Drinking several times a week	61 (21.4%)	
Occasional drinker	70 (24.6%)	
Non-drinker	71 (24.9%)	
**COVID-19 severity**		
Asymptomatic	6 (2.1%)	0
Mild	64 (22.5%)	0
Moderate I	139 (48.8%)	0
Moderate II	53 (18.6%)	0
Severe	22 (7.7%)	0
Unknown	1 (0.4%)	0
**Underlying disease**		
Hypertension	84 (29.5%)	0
Diabetes	51 (17.9%)	0
Dyslipidemia	43 (15.1%)	0
Chronic heart disease	13 (4.6%)	0
Respiratory disease	14 (4.9%)	0
Cerebrovascular disease	10 (3.5%)	0
Solid malignancy	4 (1.4%)	0
Hematological malignancy	2 (0.7%)	0
Collagen disease	3 (1.1%)	0
HIV/AIDS	2 (0.7%)	0
Vascular lesion of limbs	4 (1.4%)	0
**Laboratory test results**		
WBC (/μL)	5170 (3840–7120)	46
Lymp (/μL)	1047.5 (734.8–1336.5)	47
PLAT (× 10^4^/μL)	20.0 (15.2–25.0)	46
Hb (g/dL)	13.8 (12.6–15.0)	46
Alb (g/dL)	3.7 (3.4–4.0)	47
AST (U/L)	30.0 (22.0–47.0)	46
ALT (U/L)	24.0 (17.0–42.5)	46
Cre (mg/dL)	0.77 (0.61–0.94)	47
LDH (U/L)	242.0 (192.0–315.5)	46
CK (U/L)	72.0 (43.0–124.0)	50
CK-MB (ng/mL)	5.0 (4.3–6.5)	279
CRP (mg/dL)	2.6 (0.6–6.5)	46
Na (mEq/L)	138.0 (136.0–140.0)	46
K (mEq/L)	3.9 (3.6–4.2)	47
D-dimer (μg/mL)	0.80 (0.60–1.27)	77
FDP (μg/mL)	4.3 (3.7–6.3)	263
Ferritin (ng/mL)	490.0 (180.0–942.0)	92
PCT (ng/mL)	0.04 (0.03–0.07)	154
KL-6 (U/mL)	247.0 (191.5–356.5)	154
PT (seconds)	10.6 (10.3–11.1)	116

AIDS, acquired immunodeficiency syndrome; Alb, albumin; ALT, alanine aminotransferase; AST, aspartate aminotransferase; CK, creatine kinase; Cre, creatinine; CRP, C-reactive protein; FDP, fibrin degradation product; HIV, human immunodeficiency virus; Hb, hemoglobin; IQR, interquartile range; K, potassium; KL-6, Krebs von den Lungen-6; LDH, lactate dehydrogenase; Lymp, lymphocytes; Na, sodium; PCT, procalcitonin; PLAT, platelet; PT, prothrombin time; WBC, white blood cell.

**Table 2:** Patients who have had at least one or more symptoms.

	Count (%)	95% CI
**All severity (N = 285)**		
Patients with at least one symptom (during COVID-19)	234 (85.3%)	80.6–89.2%
Patients with at least one symptom (At the time of questionnaire)	160 (56.1%)	50.2–62.0%
**Mild Group (N = 70)**		
Patients with at least one symptom (during COVID-19)	60 (85.7%)	75.3–92.9%
Patients with at least one symptom (At the time of questionnaire)	37 (52.9%)	40.6–64.9%
**Severe Group (N = 214)**		
Patients with at least one symptom (during COVID-19)	182 (85.1%)	79.6–89.5%
Patients with at least one symptom (At the time of questionnaire)	123 (57.5%)	50.6–64.2%

CI, confidence interval; COVID-19, Mild Group, Asymptomatic and Mild; Severe Group, Moderate I/II and Severe coronavirus disease 2019.

Figure 2 shows the distribution of sequelae and their severity at the time of answering the questionnaire, and these results are shown in more detail in the supplementary tables. The prevalence of each symptom was as follows: sputum production (21%), fatigue (10.4%), dyspnea (16.2%), anosmia (12.0%), alopecia (17.1%), concentration problems (15.5%), memory problems (16.7%), joint pain (26.6%), red eyes (16.6%), and diarrhea (12.5%). The severity score was 3–4 in > 10% of participants who reported these symptoms, which is likely to affect their QOL (Fig. 2a, Supplementary Table S1). In the Mild group, > 10% of patients with alopecia (25.0%), concentration problems (12.5%), memory problems (11.1%), sleeplessness (22.2%), and joint pain (25.0%) had a symptom severity score of 3–4 (Fig. 2b, Supplementary Table S2). In the Severe group, the prevalence of each symptom was as follows: cough (10.0%), sputum production (22.2%), fatigue (11.1%), dyspnea (17.7%), anosmia (15.0%), lack of appetite (10.0%), alopecia (14.3%), concentration problems (16.2%), memory problems (17.8%), sleeplessness (26.9%), joint pain (26.9%), red eyes (20.0%), and diarrhea (12.5%) (Fig. 2c, Supplemental Table S3).

**Figure 3:**
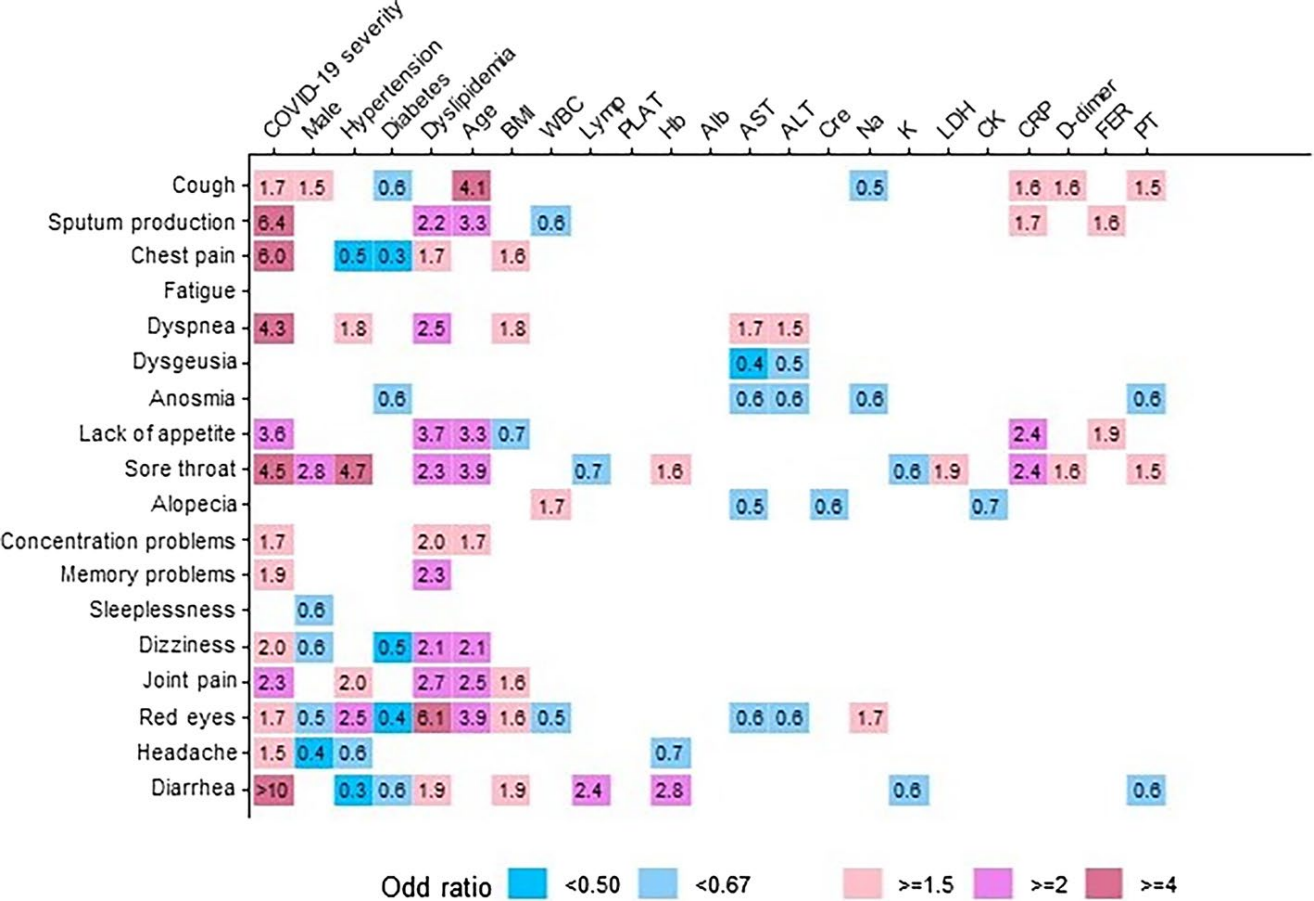
Risk factors for each persistent symptom using univariable ordinal logistic regression.

In regression analysis, risk factors for each persisting symptom were analyzed. The numbers indicate the OR between each factor (horizontal axis) and the remaining symptoms (vertical axis). Strong associations (OR ≥ 4) were observed for age and cough, COVID-19 severity and sputum production/chest pain/dyspnea/sore throat/diarrhea/hypertension and sore throat, and dyslipidemia and red eyes. No association was observed between COVID-19 severity and fatigue, dysgeusia, anosmia, alopecia, or sleeplessness (Fig. 3). The time to recovery from each symptom among participants whose symptoms resolved prior to is shown in Tables 3, 4 and 5. The following symptoms tended to persist (> 50% present for ≥ 1 year): fatigue, alopecia, concentration problems, memory problems, sleeplessness, dizziness, joint pain, red eyes, headache, and diarrhea (Table 3). the findings were similar regardless of the initial disease severity. The results of participants in the Mild group (Table 4) were similar to those of participants in the Severe group (Table 5), except for diarrhea which only persisted in patients with severe disease.

**Table 3:** Time to recovery of each symptom in all participants.

	N	Time to recovery			
		Within 3 months (%)	3 to 6 months (%)	6 months to 1 year (%)	More than 1 year (%)
Cough	75	65.3	2.7	0.0	32.0
Sputum production	56	60.7	3.6	3.2	32.5
Chest pain	48	52.1	8.3	3.0	36.5
Fatigue	100	35.0	3.0	4.4	57.6
Dyspnea	87	44.8	8.0	7.4	39.7
Dysgeusia	74	58.1	1.4	2.3	38.3
Anosmia	70	52.9	2.9	8.4	35.9
Lack of appetite	62	53.2	9.7	3.1	34.0
Sore throat	37	54.1	8.1	0.0	37.8
Alopecia	72	15.3	8.3	16.4	60.0
Concentration problems	62	16.1	9.7	3.5	70.7
Memory problems	64	9.4	6.2	0.0	84.4
Sleeplessness	45	15.6	6.7	0.0	77.8
Dizziness	32	25.0	9.4	6.0	59.7
Joint pain	43	27.9	0.0	6.6	65.5
Red eyes	15	6.7	0.0	37.3	56.0
Headache	38	23.7	2.6	0.0	73.7
Diarrhea	14	35.7	0.0	0.0	64.3

**Table 4:** Time to recovery of each symptom in Mild Group.

	N	Time to recovery			
		Within 3 months (%)	3 to 6 months (%)	6 months to 1 year (%)	More than 1 year (%)
Cough	17	76.5	0.0	0.0	23.5
Sputum production	10	90.0	0.0	0.0	10.0
Chest pain	4	75.0	0.0	0.0	25.0
Fatigue	22	36.4	4.5	0.0	59.1
Dyspnea	11	54.5	18.2	0.0	27.3
Dysgeusia	19	63.2	5.3	0.0	31.6
Anosmia	19	57.9	10.5	7.9	23.7
Lack of appetite	9	77.8	0.0	0.0	22.2
Sore throat	5	80.0	0.0	0.0	20.0
Alopecia	16	18.8	0.0	10.2	71.1
Concentration problems	11	27.3	0.0	0.0	72.7
Memory problems	10	10.0	0.0	0.0	90.0
Sleeplessness	11	0.0	18.2	0.0	81.8
Dizziness	5	40.0	0.0	0.0	60.0
Joint pain	7	42.9	0.0	0.0	57.1
Red eyes	2	0.0	0.0	0.0	100.0
Headache	9	44.4	0.0	0.0	55.6
Diarrhea	2	100.0	0.0	0.0	0.0

**Table 5:** Time to recovery of each symptom in Severe Group.

	N	Time to recovery			
		Within 3 months (%)	3 to 6 months (%)	6 months to 1 year (%)	More than 1 year (%)
Cough	58	62.1	3.4	0.0	34.5
Sputum production	46	54.3	4.3	3.8	37.5
Chest pain	44	50.0	9.1	3.4	37.5
Fatigue	78	34.6	2.6	6.3	56.5
Dyspnea	76	43.4	6.6	8.3	41.7
Dysgeusia	55	56.4	0.0	3.1	40.5
Anosmia	51	51.0	0.0	8.7	40.4
Lack of appetite	53	49.1	11.3	3.6	36.0
Sore throat	32	50.0	9.4	0.0	40.6
Alopecia	56	14.3	10.7	18.8	56.2
Concentration problems	51	13.7	11.8	4.4	70.1
Memory problems	54	9.3	7.4	0.0	83.3
Sleeplessness	34	20.6	2.9	0.0	76.5
Dizziness	27	22.2	11.1	8.3	58.3
Joint pain	36	25.0	0.0	8.3	66.7
Red eyes	13	7.7	0.0	36.9	55.4
Headache	29	17.2	3.4	0.0	79.3
Diarrhea	12	25.0	0.0	0.0	75.0

## Discussion

To date, only one small survey has investigated the actual condition of Long COVID or COVID-19 sequelae in the Japanese population^19^, and Long COVID is poorly understood in Japan. Furthermore, to the best of our knowledge, only a few studies have conducted a large-scale investigation globally regarding COVID-19 sequelae, and no study has examined the risk factors for each persisting symptom separately. We assessed the duration and severity of each persisting symptom; and identified the sequelae that have long-term effects on the QOL.

Of the study participants, 56.1% had one or more sequelae at the time of completing the questionnaire, approximately 1 year (median) post-infection. According to a report from Italy, 87.4% of infected individuals had one or more sequelae 2 months after infection^16^. Similarly, a report from China suggested that some symptoms persisted in 76% of infected individuals 6 months post-infection^18^. Considering the long duration of symptoms in our study compared to these studies, a slightly lower prevalence of sequelae may be reasonable. If the severity of the symptoms is not considered, even > 50% of infected participants with mild COVID-19 has one or more sequelae (Table 2). Furthermore, the prevalence of Long COVID was similar in the Mild and Severe groups, showing that Long COVID is not dependent on the initial disease severity. The distribution of symptoms differed between the acute phase and 1-year post-infection. This tendency was similar regardless of COVID-19 severity. Comparing the distribution of sequelae at the time of answering the questionnaire in the Mild group, Severe group, and overall, the distribution was similar; fatigue, alopecia, concentration problems, memory problems, and sleeplessness persisted among ≥ 10% of participants. In a previous report, fatigue, cough, red eyes, and dyspnea were observed in > 30% of participants 2–3 weeks after COVID-19 onset^15^. Similarly, in a survey conducted 4–8 weeks post-COVID-19 onset, fatigue, dyspnea, post-traumatic stress disorder (PTSD), anxiety and depression, and concentration problems persisted in > 30% of patients in the intensive care unit and general ward^17^. Furthermore, in a study of patients 6 months after COVID-19 onset, fatigue was the most common persistent symptom, persisting among ≥ 60% of participants, and sleep disorders and alopecia persisted among ≥ 20% of participants^18^. Consistent with previous studies^16,22,23^, fatigue was the most common persistent symptom in our study. Other symptoms varied among studies—they might be influenced by the time of the study, the epidemic strain of SARS-CoV-2, and ethnicity.

Regarding the effects of each persistent symptom on the QOL, we considered the symptom severity score of 3–4 to be the most influential. In both Mild and Severe groups, many participants had severe symptoms of alopecia, concentration problems, memory problems, sleeplessness, and joint pain (Supplementary Tables S2 and S3). Symptoms that strongly manifested in the Mild group were also strongly exhibited in the Severe group. Additionally, in the Severe group, persistent cough, sputum production, fatigue, dyspnea, lack of appetite, red eyes, and diarrhea affected the QOL. Thus, more severe COVID-19 is associated with a greater diversity of sequelae affecting the QOL.

Moreno-Pérez et al.^22^ analyzed risk factors for COVID-19 sequelae and detected no significant risk factors. This may be because the investigators did not consider the risk factors separately for each symptom Therefore, we analyzed the factors related to each persistent symptom. Persistent sputum production and dyspnea were strongly related to the severity of COVID-19 (Fig. 3). Further, although the OR was low for cough, it was one of the most common respiratory symptoms of COVID-19 and was associated with the severity of the disease. The higher the severity of COVID-19, the greater the organic damage caused to the lungs, and the severity may be strongly associated with these respiratory sequelae. According to previous studies, more severe COVID-19 is associated with a decrease in the residual air volume and diffusivity^18^. Furthermore, people with dyspnea have low forced vital capacity, low forced expiratory volume in 1 s and diffusing capacity for carbon monoxide, and restrictive ventilatory patterns^24^. Greater severity and severe acute organic lung damage may cause the persistence of respiratory symptoms. In addition to respiratory symptoms, chest pain, sore throat, and diarrhea were closely associated with COVID-19 severity and sequelae. The association between gastrointestinal symptoms and COVID-19 may be due to disturbances in the immune system and intestinal flora^25^, and diarrhea has previously been reported to be associated with COVID-19 severity^26^. Patients with more severe COVID-19 may develop sequelae because of long-term immune system activation and disturbances in the intestinal flora. Myocardial inflammation has been reported to persist for up to 71 days post-COVID-19 and may be associated with long-term chest pain^27^. It is unknown whether sore throat is caused by Long COVID. Hence, further research is needed to explain the direct causal relationship between diarrhea and sore throat as sequelae and COVID-19 severity.

Fatigue, dysgeusia, anosmia, alopecia, and sleeplessness as sequelae were not related to COVID-19 severity. It has previously been reported that fatigue is not associated with COVID-19 severity^28^. Thus, individuals such as, young individuals, those without any underlying disease, vaccinated individuals, and those with a previous history of COVID-19, may experience these sequelae, despite being at low risk of developing severe disease. Therefore, preventing infection is important until there is treatment available for specific COVID-19 sequelae.

In the context of recovery time from symptoms of fatigue, alopecia, and sleeplessness, it was observed that many patients (over 50%) did not recover from their symptoms even after ≥ 1 year of SARS-CoV-2 infection. These symptoms were unrelated to COVID-19 severity, suggesting that even mildly ill individuals may experience sequelae over a long period. Hence, attention should be paid to sequelae of the Omicron variant of SARS-CoV-2, which has been dominant in 2022 and is considered highly infectious and mildly severe^29–31^.

Our study has some limitations. First, it was a cross-sectional study; thus, the time from disease occurrence to investigation varied among patients, and patients could have been infected with different viral strains or variants. We could not investigate the infecting strain in each patient. Second, selection bias could affect the participant’s willingness to participate, and recall bias may be involved in the severity of sequelae symptoms^32^. People with Long COVID are more likely to participate in questionnaire studies about COVID-19 sequelae. Conversely, people without sequelae are more likely to be uninterested in the study and, consequently, may not have participated. Further, we may have overestimated the prevalence of COVID-19 sequelae. Accuracy of memory and recall may also vary among participants for acute symptoms. Finally, although we obtained new findings on risk factors for COVID-19 sequelae, it is difficult to propose countermeasures to prevent the sequelae of COVID-19 besides infection prevention and control.

In conclusion, this study revealed a high prevalence of sequelae approximately 1 year after COVID-19. Fatigue, dysgeusia, anosmia, alopecia, and sleeplessness as sequelae of COVID-19 can affect the QOL, even in individuals with asymptomatic or mild disease, and the sequelae are prolonged. Thus, preventing COVID-19 is important even among individuals who are not at the risk of severe disease.

## Supplementary Information

Supplementary Tables.

## Data Availability

The datasets used and/or analyzed during the current study available from the corresponding author on reasonable request.
