# Supplementary material for: A cross-sectional, multicenter survey of the prevalence and risk factors for Long COVID
Source: Sci Rep. 2022 Dec 27;12:22413. doi: 10.1038/s41598-022-25398-6 (PMC9793373; doi:10.1038/s41598-022-25398-6)
Supplement: Supplementary file 1 — Supplementary Tables. [file 41598_2022_25398_MOESM1_ESM.docx]

**A cross-sectional, multicenter survey of the prevalence and risk factors for Long COVID**

Waki Imoto^1,2,3^, Koichi Yamada^1,2,3^, Ryota Kawai^4^, Takumi Imai^4^, Kengo Kawamoto^5^, Masato Uji^5^, Hidenori Kanda^6^, Minoru Takada^7^, Yoshiteru Ohno^8^, Hiroshi Ohtani^9^, Manami Kono^10^, Atsuhito Hikiishi^10^, Yosuke Eguchi^10^, Hiroki Namikawa^11^, Tomoya Kawaguchi^12^, Hiroshi Kakeya^1,2,3,13,*^

^1^Department of Infection Control Science, Osaka Metropolitan University Graduate School of Medicine, 1-4-3, Asahi-machi, Abeno-ku, Osaka 545-8585, Japan

^2^Department of Infectious Disease Medicine, Osaka Metropolitan University Hospital, 1-5-7 Asahi-machi, Abeno-ku, Osaka 545-8586, Japan

^3^Department of Infection Control and Prevention, Osaka Metropolitan University Hospital, 1-5-7 Asahi-machi, Abeno-ku, Osaka 545-8586, Japan

^4^Department of Medical Statistics, Osaka Metropolitan University Graduate School of Medicine, 1-4-3, Asahi-machi, Abeno-ku, Osaka 545-8585, Japan

^5^Department of Respiratory Medicine, Osaka City Juso Hospital, 2-12-27, Nonakakita, Yodogawa-ku, Osaka 532-0034, Japan

^6^Department of Internal Medicine, KINSHUKAI, Hanwa The Second Hospital, 2-4-5, Abiko Nishi, Sumiyoshi-ku, Osaka 558-0015, Japan

^7^Department of Internal Medicine, KINSHUKAI, Hanwa The Second Senboku Hospital, 3176, Fukai Kitamachi, Naka-ku, Sakai-city, Osaka 559-8271, Japan

^8^Department of Internal Medicine, Ohno Memorial Hospital, 1-26-10, Minamihorie, Nishi-ku, Osaka 550-0015, Japan

^9^Department of Surgery, Ohno Memorial Hospital, 1-26-10, Minamihorie, Nishi-ku, Osaka 550-0015, Japan

^10^Department of Respiratory Medicine, BellLand general Hospital, 500-3, Higashiyama, Naka-ku, Sakai-shi, Osaka, 599-8247, Japan

^11^Department of Medical Education and General Practice, Osaka Metropolitan University Graduate School of Medicine, 1-4-3, Asahi-machi, Abeno-ku, Osaka 545-8585, Japan

^12^Department Respiratory Medicine, Osaka Metropolitan University Graduate School of Medicine, 1-4-3, Asahi-machi, Abeno-ku, Osaka 545-8585, Japan

^13^Research Center for Infectious Disease Sciences, Osaka Metropolitan University Graduate School of Medicine, 1-4-3, Asahi-machi, Abeno-ku, Osaka 545-8585, Japan

***Corresponding author:**

Hiroshi Kakeya, MD, PhD

Department of Infection Control Science

Graduate School of Medicine, Osaka Metropolitan University

1-4-3 Asahi-machi, Abeno-ku, Osaka 545-8585, Japan

Telephone: +81-6-6645-3784

Fax: +81-6-6646-6056

E-mail: [kakeya-ngs@umin.ac.jp](mailto:kakeya-ngs@umin.ac.jp/kakeya@med.osaka-cu.ac.jp); [kakeya@med.osaka-cu.ac.jp](mailto:kakeya-ngs@umin.ac.jp/kakeya@med.osaka-cu.ac.jp)

Supplementary Table S1. Distribution of Long COVID symptoms in all participants (N = 285)

|  | **Asymptomatic**  **Severity 0** | **Symptomatic** | | | | |
| --- | --- | --- | --- | --- | --- | --- |
|  |  | **All (Severity 1–4)** | **Severity 1** | **Severity 2** | **Severity 3** | **Severity 4** |
| Symptoms | Count | Count | Count (%*) | Count (%*) | Count (%*) | Count (%*) |
| Cough | 261 | 24 | 19 (79.2%) | 3 (12.5%) | 2 (8.3%) | 0 (0.0%) |
| Sputum production | 266 | 19 | 12 (63.2%) | 3 (15.8%) | 2 (10.5%) | 2 (10.5%) |
| Chest pain | 267 | 18 | 15 (83.3%) | 2 (11.1%) | 1 (5.6%) | 0 (0.0%) |
| Fatigue | 227 | 58 | 36 (62.1%) | 16 (27.6%) | 3 (5.2%) | 3 (5.2%) |
| Dyspnea | 248 | 37 | 24 (64.9%) | 7 (18.9%) | 4 (10.8%) | 2 (5.4%) |
| Dysgeusia | 258 | 27 | 20 (74.1%) | 7 (25.9%) | 0 (0.0%) | 0 (0.0%) |
| Anosmia | 260 | 25 | 17 (68.0%) | 5 (20.0%) | 0 (0.0%) | 3 (12.0%) |
| Lack of appetite | 263 | 22 | 16 (72.7%) | 4 (18.2%) | 1 (4.5%) | 1 (4.5%) |
| Sore throat | 271 | 14 | 9 (64.3%) | 4 (28.6%) | 1 (7.1%) | 0 (0.0%) |
| Alopecia | 238 | 47 | 29 (61.7%) | 10 (21.3%) | 6 (12.8%) | 2 (4.3%) |
| Concentration problems | 240 | 45 | 24 (53.3%) | 14 (31.1%) | 5 (11.1%) | 2 (4.4%) |
| Memory problems | 231 | 54 | 26 (48.1%) | 19 (35.2%) | 7 (13.0%) | 2 (3.7%) |
| Sleeplessness | 250 | 35 | 14 (40.0%) | 12 (34.3%) | 8 (22.9%) | 1 (2.9%) |
| Dizziness | 265 | 20 | 10 (50.0%) | 10 (50.0%) | 0 (0.0%) | 0 (0.0%) |
| Joint pain | 255 | 30 | 13 (43.3%) | 9 (30.0%) | 4 (13.3%) | 4 (13.3%) |
| Red eyes | 273 | 12 | 9 (75.0%) | 1 (8.3%) | 1 (8.3%) | 1 (8.3%) |
| Headache | 257 | 28 | 17 (60.7%) | 11 (39.3%) | 0 (0.0%) | 0 (0.0%) |
| Diarrhea | 277 | 8 | 5 (62.5%) | 2 (25.0%) | 1 (12.5%) | 0 (0.0%) |
| *Distribution ratio among those with each symptom | | | | | | |

Supplementary Table S2. Distribution of Long COVID symptoms in the Mild group (N = 70)

|  | **Asymptomatic**  **Severity 0** | **Symptomatic** | | | | |
| --- | --- | --- | --- | --- | --- | --- |
|  |  | **All (Severity 1–4)** | **Severity 1** | **Severity 2** | **Severity 3** | **Severity 4** |
| Symptoms | Count | Count | Count (%*) | Count (%*) | Count (%*) | Count (%*) |
| Cough | 66 | 4 | 4 (100.0%) | 0 (0.0%) | 0 (0.0%) | 0 (0.0%) |
| Sputum production | 69 | 1 | 1 (100.0%) | 0 (0.0%) | 0 (0.0%) | 0 (0.0%) |
| Chest pain | 69 | 1 | 1 (100.0%) | 0 (0.0%) | 0 (0.0%) | 0 (0.0%) |
| Fatigue | 57 | 13 | 11 (84.6%) | 1 (7.7%) | 0 (0.0%) | 1 (7.7%) |
| Dyspnea | 67 | 3 | 3 (100.0%) | 0 (0.0%) | 0 (0.0%) | 0 (0.0%) |
| Dysgeusia | 64 | 6 | 4 (66.7%) | 2 (33.3%) | 0 (0.0%) | 0 (0.0%) |
| Anosmia | 65 | 5 | 3 (60.0%) | 2 (40.0%) | 0 (0.0%) | 0 (0.0%) |
| Lack of appetite | 68 | 2 | 2 (100.0%) | 0 (0.0%) | 0 (0.0%) | 0 (0.0%) |
| Sore throat | 69 | 1 | 1 (100.0%) | 0 (0.0%) | 0 (0.0%) | 0 (0.0%) |
| Alopecia | 58 | 12 | 7 (58.3%) | 2 (16.7%) | 2 (16.7%) | 1 (8.3%) |
| Concentration problems | 62 | 8 | 7 (87.5%) | 0 (0.0%) | 0 (0.0%) | 1 (12.5%) |
| Memory problems | 61 | 9 | 6 (66.7%) | 2 (22.2%) | 0 (0.0%) | 1 (11.1%) |
| Sleeplessness | 61 | 9 | 5 (55.6%) | 2 (22.2%) | 2 (22.2%) | 0 (0.0%) |
| Dizziness | 67 | 3 | 2 (66.7%) | 1 (33.3%) | 0 (0.0%) | 0 (0.0%) |
| Joint pain | 66 | 4 | 1 (25.0%) | 2 (50.0%) | 0 (0.0%) | 1 (25.0%) |
| Red eyes | 68 | 2 | 2 (100.0%) | 0 (0.0%) | 0 (0.0%) | 0 (0.0%) |
| Headache | 65 | 5 | 2 (40.0%) | 3 (60.0%) | 0 (0.0%) | 0 (0.0%) |
| Diarrhea | 70 | 0 | 0 (0.0%) | 0 (0.0%) | 0 (0.0%) | 0 (0.0%) |
| *Distribution ratio among those with each symptom | | | | | | |

Supplementary Table S3. Distribution of Long COVID symptoms in the Severe group (N =214)

|  | **Asymptomatic Severity 0** | **Symptomatic** | | | | |
| --- | --- | --- | --- | --- | --- | --- |
|  |  | **All (Severity 1–4)** | **Severity 1** | **Severity 2** | **Severity 3** | **Severity 4** |
| Symptoms | Count | Count | Count (%*) | Count (%*) | Count (%*) | Count (%*) |
| Cough | 194 | 20 | 15 (75.0%) | 3 (15.0%) | 2 (10.0%) | 0 (0.0%) |
| Sputum production | 196 | 18 | 11 (61.1%) | 3 (16.7%) | 2 (11.1%) | 2 (11.1%) |
| Chest pain | 197 | 17 | 14 (82.4%) | 2 (11.8%) | 1 (5.9%) | 0 (0.0%) |
| Fatigue | 169 | 45 | 25 (55.6%) | 15 (33.3%) | 3 (6.7%) | 2 (4.4%) |
| Dyspnea | 180 | 34 | 21 (61.8%) | 7 (20.6%) | 4 (11.8%) | 2 (5.9%) |
| Dysgeusia | 193 | 21 | 6 (76.2%) | 5 (23.8%) | 0 (0.0%) | 0 (0.0%) |
| Anosmia | 194 | 20 | 4 (70.0%) | 3 (15.0%) | 0 (0.0%) | 3 (15.0%) |
| Lack of appetite | 194 | 20 | 4 (70.0%) | 4 (20.0%) | 1 (5.0%) | 1 (5.0%) |
| Sore throat | 201 | 13 | 8 (61.5%) | 4 (30.8%) | 1 (7.7%) | 0 (0.0%) |
| Alopecia | 179 | 35 | 22 (62.9%) | 8 (22.9%) | 4 (11.4%) | 1 (2.9%) |
| Concentration problems | 177 | 37 | 17 (45.9%) | 14 (37.8%) | 5 (13.5%) | 1 (2.7%) |
| Memory problems | 169 | 45 | 20 (44.4%) | 17 (37.8%) | 7 (15.6%) | 1 (2.2%) |
| Sleeplessness | 188 | 26 | 9 (34.6%) | 10 (38.5%) | 6 (23.1%) | 1 (3.8%) |
| Dizziness | 197 | 17 | 8 (47.1%) | 9 (52.9%) | 0 (0.0%) | 0 (0.0%) |
| Joint pain | 188 | 26 | 12 (46.2%) | 7 (26.9%) | 4 (15.4%) | 3 (11.5%) |
| Red eyes | 204 | 10 | 7 (70.0%) | 1 (10.0%) | 1 (10.0%) | 1 (10.0%) |
| Headache | 191 | 23 | 15 (65.2%) | 8 (34.8%) | 0 (0.0%) | 0 (0.0%) |
| Diarrhea | 206 | 8 | 5 (62.5%) | 2 (25.0%) | 1 (12.5%) | 0 (0.0%) |
| *Distribution ratio among those with each symptom | | | | | | |

Supplementary Table S4-1. Hazard ratios and 95% confidence intervals of Figure 3.

|  | COVID-19 severity | Male | Hypertension | Diabetes | Dyslipidemia | Age | BMI | WBC | Lymp | PLAT | Hb | Alb |
| --- | --- | --- | --- | --- | --- | --- | --- | --- | --- | --- | --- | --- |
| Cough | 1.7 (0.6 to 5.3) | 1.5 (0.6 to 3.7) | 1.3 (0.5 to 3.0) | 0.6 (0.2 to 2.2) | 1.2 (0.4 to 3.6) | 4.1 (1.7 to 10.1) | 0.7 (0.4 to 1.3) | 1.1 (0.6 to 2.0) | 0.9 (0.5 to 1.7) | 1.1 (0.7 to 1.6) | 0.7 (0.4 to 1.3) | 0.8 (0.5 to 1.3) |
| Sputum  production | 6.4 (0.8 to 48.8) | 0.8 (0.3 to 2.1) | 1.1 (0.4 to 3.0) | 1.2 (0.4 to 3.8) | 2.2 (0.8 to 6.5) | 3.3 (1.3 to 8.5) | 1.3 (0.7 to 2.3) | 0.6 (0.3 to 1.2) | 0.8 (0.4 to 1.4) | 1.2 (0.8 to 1.8) | 1.2 (0.6 to 2.3) | 0.8 (0.5 to 1.3) |
| Chest pain | 6.0 (0.8 to 45.9) | 1.0 (0.4 to 2.5) | 0.5 (0.1 to 1.6) | 0.3 (0.0 to 2.0) | 1.7 (0.5 to 5.4) | 1.1 (0.5 to 2.4) | 1.6 (0.9 to 2.8) | 1.0 (0.6 to 2.0) | 1.0 (0.5 to 1.7) | 1.2 (0.8 to 1.8) | 1.4 (0.7 to 2.9) | 1.0 (0.8 to 1.3) |
| Fatigue | 1.2 (0.6 to 2.5) | 0.7 (0.4 to 1.3) | 1.1 (0.6 to 2.1) | 0.9 (0.4 to 2.0) | 1.5 (0.7 to 3.1) | 1.3 (0.8 to 2.0) | 1.0 (0.7 to 1.4) | 0.7 (0.5 to 1.1) | 1.3 (0.8 to 1.9) | 0.9 (0.7 to 1.2) | 1.0 (0.7 to 1.5) | 1.0 (0.8 to 1.2) |
| Dyspnea | 4.3 (1.3 to 14.5) | 1.0 (0.5 to 2.0) | 1.8 (0.9 to 3.6) | 0.7 (0.3 to 1.9) | 2.5 (1.1 to 5.5) | 1.3 (0.7 to 2.3) | 1.8 (1.2 to 2.7) | 1.1 (0.7 to 1.8) | 1.1 (0.7 to 1.7) | 0.9 (0.7 to 1.3) | 1.0 (0.6 to 1.6) | 0.9 (0.7 to 1.2) |
| Dysgeusia | 1.2 (0.4 to 3.0) | 0.9 (0.4 to 2.1) | 1.0 (0.4 to 2.4) | 1.0 (0.4 to 2.8) | 1.0 (0.3 to 3.0) | 1.2 (0.6 to 2.4) | 1.1 (0.6 to 1.8) | 0.8 (0.4 to 1.4) | 1.0 (0.6 to 1.7) | 0.8 (0.5 to 1.3) | 1.1 (0.6 to 1.9) | 1.0 (0.7 to 1.3) |
| Anosmia | 1.3 (0.5 to 3.7) | 0.7 (0.3 to 1.5) | 1.0 (0.4 to 2.4) | 0.6 (0.2 to 2.1) | 0.7 (0.2 to 2.5) | 0.9 (0.5 to 1.7) | 0.7 (0.4 to 1.3) | 0.7 (0.4 to 1.2) | 1.3 (0.7 to 2.5) | 0.9 (0.5 to 1.3) | 1.2 (0.7 to 2.2) | 1.0 (0.8 to 1.3) |
| Lack of  appetite | 3.6 (0.8 to 15.6) | 0.9 (0.4 to 2.2) | 0.9 (0.3 to 2.4) | 1.0 (0.3 to 3.1) | 3.7 (1.4 to 9.4) | 3.3 (1.3 to 8.0) | 0.7 (0.3 to 1.3) | 1.1 (0.6 to 2.0) | 0.9 (0.5 to 1.5) | 1.0 (0.7 to 1.6) | 0.7 (0.4 to 1.3) | 0.8 (0.5 to 1.2) |
| Sore throat | 4.5 (0.6 to 35.1) | 2.8 (0.8 to 10.4) | 4.7 (1.5 to 14.4) | 1.3 (0.3 to 4.7) | 2.3 (0.7 to 7.8) | 3.9 (1.2 to 12.5) | 1.4 (0.7 to 2.6) | 0.9 (0.4 to 1.8) | 0.7 (0.4 to 1.2) | 1.0 (0.6 to 1.7) | 1.6 (0.7 to 3.6) | 0.7 (0.4 to 1.2) |
| Alopecia | 0.9 (0.5 to 1.9) | 0.8 (0.4 to 1.4) | 1.2 (0.6 to 2.3) | 1.3 (0.6 to 2.8) | 1.1 (0.5 to 2.6) | 0.9 (0.6 to 1.6) | 0.9 (0.6 to 1.4) | 1.7 (1.1 to 2.7) | 1.2 (0.8 to 2.0) | 1.1 (0.8 to 1.5) | 1.1 (0.7 to 1.7) | 0.9 (0.6 to 1.2) |
| Concentration  problems | 1.7 (0.8 to 3.8) | 0.8 (0.4 to 1.4) | 1.2 (0.6 to 2.4) | 0.9 (0.4 to 2.1) | 2.0 (0.9 to 4.3) | 1.7 (0.9 to 2.9) | 1.0 (0.6 to 1.5) | 1.0 (0.7 to 1.6) | 1.2 (0.7 to 1.8) | 1.0 (0.7 to 1.3) | 1.1 (0.7 to 1.7) | 0.9 (0.6 to 1.2) |
| Memory  problems | 1.9 (0.9 to 4.0) | 0.8 (0.4 to 1.5) | 0.8 (0.4 to 1.6) | 1.0 (0.5 to 2.2) | 2.3 (1.1 to 4.8) | 1.4 (0.9 to 2.4) | 1.2 (0.9 to 1.8) | 0.8 (0.5 to 1.2) | 1.0 (0.6 to 1.5) | 0.9 (0.6 to 1.2) | 1.0 (0.6 to 1.4) | 0.9 (0.6 to 1.2) |
| Sleeplessness | 1.0 (0.4 to 2.2) | 0.6 (0.3 to 1.2) | 0.7 (0.3 to 1.6) | 0.7 (0.3 to 2.0) | 1.2 (0.5 to 3.1) | 1.1 (0.6 to 2.0) | 1.0 (0.6 to 1.6) | 1.2 (0.7 to 1.9) | 1.0 (0.6 to 1.6) | 0.9 (0.6 to 1.3) | 0.9 (0.6 to 1.5) | 0.7 (0.4 to 1.2) |
| Dizziness | 2.0 (0.6 to 6.9) | 0.6 (0.2 to 1.5) | 1.0 (0.4 to 2.8) | 0.5 (0.1 to 2.2) | 2.1 (0.7 to 6.1) | 2.1 (0.9 to 4.9) | 0.8 (0.4 to 1.5) | 1.0 (0.5 to 1.8) | 1.5 (0.7 to 2.9) | 1.0 (0.6 to 1.5) | 0.9 (0.5 to 1.8) | 0.9 (0.7 to 1.3) |
| Joint pain | 2.3 (0.8 to 6.7) | 0.9 (0.4 to 1.8) | 2.0 (0.9 to 4.2) | 0.7 (0.2 to 2.0) | 2.7 (1.1 to 6.3) | 2.5 (1.2 to 5.2) | 1.6 (1.0 to 2.6) | 1.2 (0.7 to 2.1) | 1.0 (0.6 to 1.7) | 1.1 (0.7 to 1.5) | 0.8 (0.5 to 1.3) | 0.8 (0.5 to 1.2) |
| Red eyes | 1.7 (0.4 to 7.9) | 0.5 (0.2 to 1.7) | 2.5 (0.8 to 8.1) | 0.4 (0.1 to 3.2) | 6.1 (1.9 to 19.8) | 3.9 (1.1 to 13.8) | 1.6 (0.9 to 3.2) | 0.5 (0.2 to 1.2) | 1.1 (0.5 to 2.4) | 1.0 (0.6 to 1.8) | 0.8 (0.4 to 1.6) | 1.1 (0.8 to 1.4) |
| Headache | 1.5 (0.6 to 4.2) | 0.4 (0.2 to 0.9) | 0.6 (0.2 to 1.6) | 0.7 (0.2 to 2.2) | 1.3 (0.4 to 3.5) | 1.1 (0.6 to 2.0) | 1.0 (0.6 to 1.7) | 1.1 (0.6 to 2.0) | 1.1 (0.6 to 1.9) | 0.8 (0.5 to 1.3) | 0.7 (0.4 to 1.1) | 1.0 (0.7 to 1.3) |
| Diarrhea | >10 (0.0 to >10) | 1.3 (0.3 to 5.4) | 0.3 (0.0 to 2.8) | 0.6 (0.1 to 5.3) | 1.9 (0.4 to 9.6) | 0.7 (0.2 to 2.3) | 1.9 (0.9 to 4.0) | 1.0 (0.4 to 2.4) | 2.4 (0.8 to 7.2) | 0.7 (0.3 to 1.6) | 2.8 (0.8 to 9.1) | 1.1 (0.8 to 1.4) |

Abbreviations: Alb, albumin; BMI, body mass index; Hb, hemoglobin; Lymp, lymphocytes; PLAT, platelets; WBC, white blood cells

Supplementary Table S4-2. Hazard ratios and 95% confidence intervals of Figure 3.

|  | AST | ALT | Cre | Na | K | LDH | CK | CRP | D-dimer | FER | PT |
| --- | --- | --- | --- | --- | --- | --- | --- | --- | --- | --- | --- |
| Cough | 1.0 (0.5 to 2.0) | 0.7 (0.4 to 1.3) | 1.4 (0.9 to 2.0) | 0.5 (0.3 to 1.0) | 1.0 (0.9 to 1.2) | 1.3 (0.7 to 2.3) | 1.0 (0.5 to 1.7) | 1.6 (0.8 to 3.6) | 1.6 (1.2 to 2.2) | 1.5 (0.7 to 3.0) | 1.5 (1.1 to 2.2) |
| Sputum production | 1.0 (0.5 to 1.9) | 0.8 (0.5 to 1.5) | 1.0 (0.6 to 1.6) | 1.0 (0.6 to 1.7) | 0.8 (0.5 to 1.3) | 1.3 (0.7 to 2.5) | 0.8 (0.4 to 1.5) | 1.7 (0.8 to 3.9) | 1.1 (0.8 to 1.6) | 1.6 (0.8 to 3.5) | 1.1 (0.7 to 1.8) |
| Chest pain | 1.0 (0.5 to 2.0) | 1.2 (0.7 to 2.2) | 1.0 (0.6 to 1.7) | 0.8 (0.5 to 1.4) | 1.0 (0.9 to 1.1) | 1.1 (0.6 to 2.2) | 1.1 (0.7 to 1.9) | 1.5 (0.7 to 3.1) | 0.8 (0.5 to 1.3) | 1.0 (0.5 to 1.9) | 1.0 (0.6 to 1.5) |
| Fatigue | 0.9 (0.6 to 1.4) | 0.9 (0.6 to 1.3) | 0.7 (0.5 to 1.1) | 1.1 (0.8 to 1.6) | 1.0 (0.9 to 1.1) | 0.7 (0.5 to 1.1) | 1.0 (0.7 to 1.4) | 0.8 (0.5 to 1.3) | 1.0 (0.8 to 1.3) | 1.1 (0.7 to 1.6) | 1.0 (0.8 to 1.4) |
| Dyspnea | 1.7 (1.0 to 2.7) | 1.5 (1.0 to 2.3) | 0.9 (0.6 to 1.4) | 0.9 (0.6 to 1.4) | 0.9 (0.6 to 1.2) | 1.4 (0.9 to 2.3) | 1.1 (0.7 to 1.7) | 1.5 (0.8 to 2.6) | 0.9 (0.7 to 1.3) | 1.0 (0.6 to 1.6) | 1.0 (0.7 to 1.4) |
| Dysgeusia | 0.4 (0.2 to 0.9) | 0.5 (0.3 to 1.0) | 0.9 (0.6 to 1.5) | 0.7 (0.5 to 1.2) | 0.8 (0.4 to 1.4) | 0.7 (0.4 to 1.3) | 1.0 (0.6 to 1.6) | 1.1 (0.6 to 2.1) | 0.9 (0.6 to 1.3) | 1.2 (0.6 to 2.1) | 0.9 (0.5 to 1.4) |
| Anosmia | 0.6 (0.3 to 1.2) | 0.6 (0.3 to 1.1) | 0.9 (0.5 to 1.5) | 0.6 (0.4 to 1.0) | 0.8 (0.4 to 1.5) | 0.7 (0.4 to 1.3) | 1.1 (0.7 to 1.9) | 1.0 (0.5 to 1.9) | 0.9 (0.6 to 1.3) | 1.0 (0.5 to 1.8) | 0.6 (0.3 to 1.2) |
| Lack of appetite | 0.9 (0.4 to 1.8) | 0.7 (0.4 to 1.3) | 0.9 (0.5 to 1.4) | 0.8 (0.5 to 1.3) | 0.9 (0.6 to 1.3) | 1.0 (0.5 to 1.9) | 0.9 (0.5 to 1.6) | 2.4 (1.0 to 5.4) | 1.3 (0.9 to 1.8) | 1.9 (1.0 to 3.8) | 0.9 (0.5 to 1.6) |
| Sore throat | 1.1 (0.5 to 2.3) | 0.9 (0.4 to 1.8) | 0.9 (0.5 to 1.6) | 1.2 (0.6 to 2.2) | 0.6 (0.3 to 1.1) | 1.9 (0.9 to 3.8) | 1.3 (0.7 to 2.2) | 2.4 (0.9 to 6.4) | 1.6 (1.1 to 2.2) | 1.4 (0.6 to 3.1) | 1.5 (1.0 to 2.3) |
| Alopecia | 0.5 (0.3 to 0.9) | 0.8 (0.5 to 1.3) | 0.6 (0.4 to 1.0) | 0.9 (0.6 to 1.3) | 1.0 (0.8 to 1.2) | 0.9 (0.6 to 1.4) | 0.7 (0.4 to 1.1) | 0.8 (0.5 to 1.3) | 1.1 (0.9 to 1.5) | 0.8 (0.5 to 1.3) | 1.3 (1.0 to 1.8) |
| Concentration problems | 0.8 (0.5 to 1.3) | 0.8 (0.5 to 1.3) | 0.8 (0.5 to 1.2) | 0.8 (0.6 to 1.2) | 0.9 (0.7 to 1.1) | 0.9 (0.5 to 1.4) | 1.0 (0.7 to 1.4) | 1.3 (0.8 to 2.1) | 1.1 (0.8 to 1.4) | 1.2 (0.7 to 1.9) | 1.1 (0.8 to 1.5) |
| Memory problems | 0.9 (0.6 to 1.4) | 0.8 (0.5 to 1.2) | 0.8 (0.5 to 1.2) | 0.8 (0.6 to 1.2) | 0.9 (0.8 to 1.2) | 1.1 (0.7 to 1.7) | 1.2 (0.8 to 1.7) | 1.3 (0.8 to 2.0) | 1.1 (0.9 to 1.4) | 1.3 (0.8 to 2.0) | 1.1 (0.8 to 1.4) |
| Sleeplessness | 0.9 (0.5 to 1.7) | 0.8 (0.5 to 1.3) | 0.8 (0.4 to 1.3) | 0.9 (0.6 to 1.3) | 0.9 (0.7 to 1.3) | 1.1 (0.6 to 1.9) | 1.0 (0.6 to 1.7) | 1.2 (0.7 to 2.1) | 1.5 (1.1 to 1.9) | 1.2 (0.7 to 2.0) | 1.0 (0.7 to 1.5) |
| Dizziness | 1.0 (0.5 to 2.1) | 1.1 (0.6 to 1.9) | 0.8 (0.5 to 1.5) | 0.7 (0.4 to 1.2) | 0.9 (0.6 to 1.4) | 1.2 (0.6 to 2.3) | 1.1 (0.6 to 1.9) | 1.2 (0.6 to 2.5) | 1.2 (0.8 to 1.6) | 1.4 (0.7 to 2.9) | 0.8 (0.4 to 1.4) |
| Joint pain | 1.0 (0.5 to 1.7) | 0.8 (0.5 to 1.3) | 0.7 (0.4 to 1.2) | 1.1 (0.7 to 1.7) | 0.9 (0.6 to 1.3) | 1.5 (0.9 to 2.5) | 1.1 (0.7 to 1.7) | 1.4 (0.8 to 2.5) | 1.3 (1.0 to 1.8) | 0.9 (0.5 to 1.5) | 1.1 (0.8 to 1.6) |
| Red eyes | 0.6 (0.2 to 1.6) | 0.6 (0.3 to 1.4) | 0.9 (0.5 to 1.7) | 1.7 (0.9 to 3.2) | 0.8 (0.5 to 1.5) | 1.0 (0.5 to 2.4) | 1.1 (0.6 to 2.2) | 1.0 (0.4 to 2.2) | 1.2 (0.8 to 1.8) | 1.0 (0.5 to 2.3) | 0.9 (0.5 to 1.8) |
| Headache | 0.9 (0.5 to 1.6) | 0.8 (0.5 to 1.4) | 0.7 (0.4 to 1.3) | 1.2 (0.8 to 1.9) | 0.8 (0.5 to 1.3) | 1.2 (0.7 to 2.2) | 1.1 (0.7 to 1.8) | 0.8 (0.5 to 1.5) | 1.2 (0.9 to 1.7) | 0.9 (0.5 to 1.5) | 0.9 (0.6 to 1.5) |
| Diarrhea | 1.4 (0.5 to 3.5) | 1.3 (0.6 to 3.0) | 1.0 (0.5 to 2.1) | 1.3 (0.6 to 2.8) | 0.6 (0.3 to 1.4) | 0.9 (0.3 to 2.4) | 0.9 (0.4 to 2.0) | 1.0 (0.4 to 2.6) | 0.7 (0.3 to 1.4) | 1.4 (0.6 to 3.8) | 0.6 (0.2 to 1.4) |

Abbreviations: ALT, alanine aminotransferase; AST, aspartate aminotransferase; CK, creatine kinase; Cre, creatinine; CRP, C-reactive protein; FER, ferritin; LDH, lactate dehydrogenase;
PT, prothrombin time
